# Supplementary material for: The BREAK study protocol: Effects of intermittent energy restriction on adaptive thermogenesis during weight loss and its maintenance
Source: PLoS One. 2023 Nov 13;18(11):e0294131. doi: 10.1371/journal.pone.0294131 (PMC10642783; doi:10.1371/journal.pone.0294131)
Supplement: S1 File — (PDF) [file pone.0294131.s002.pdf]

COMISSÃO DE ÉTICA

PARECER Nº 31/2021/CEFCNAUP/2021

Título do Projeto:

"Efeitos da restrição energética intermitente na termogénese adaptativa e no sucesso da manutenção do peso perdido".

Submetido por:

Filipa Maria Teresa Cortez Afonso Faria, estudante do Programa Doutoral em Nutrição Clínica da Faculdade de Ciências da Nutrição e Alimentação da Universidade do Porto.

O projeto insere-se nos seus trabalhos de doutoramento, e tem como orientador o Prof. Doutor Vítor Hugo Teixeira (Faculdade de Ciências da Nutrição e Alimentação da Universidade do Porto) e coorientadora a Prof. Doutora Analiza Mónica Silva (Faculdade de Motricidade Humana da Universidade de Lisboa).

Instituições envolvidas no estudo:

Faculdade de Ciências da Nutrição e Alimentação da Universidade do Porto, Faculdade de Motricidade Humana da Universidade de Lisboa e clínica privada Farmodiética S.A.

Relator:

Teresa Amaral

Objetivos do estudo

A requerente pretende saber se a restrição energética intermitente (duas semanas alternadas com uma semana de balanço energético neutro), quando comparada com restrição energética contínua, resulta numa maior perda de peso e de massa gorda, na atenuação da perda de massa isenta de gordura, na menor termogénese adaptativa e em melhor perfil metabólico.

Pertinência e conceção do estudo

A realização do estudo está justificada e os aspetos metodológicos estão bem descritos. É proposta a realização de um estudo de desenho experimental com dois grupos paralelos, em que participarão 74 mulheres adultas obesas e idades compreendidas entre os 20 e os 45 anos, divididas aleatoriamente pelos grupos em estudo. O grupo de intervenção será submetido a restrição energética intermitente e o grupo de controlo fará restrição energética contínua.

O sucesso da manutenção do peso perdido será avaliado numa fase posterior de balanço energético neutro.

A divulgação deste estudo será realizada através do registo no https://clinicaltrials.gov/, nos media e em redes sociais.

Benefícios/riscos

Os requerentes identificaram claramente os possíveis benefícios desta intervenção, que serão a perda de peso, a otimização da composição corporal, a atenuação da termogénese adaptativa, as melhorias no perfil metabólico, o bem-estar e o aumento da autoconfiança associadas à perda de peso e a aprendizagem e adoção de um estilo de vida saudável.

São referidos os eventuais riscos/desconfortos:

1. utilização de máscara durante a calorimetria indireta;
2. recolha de 5 mL de sangue para os doseamentos séricos de T3 e T4 livre, de insulina, de leptina e de cortisol;
3. utilização de um acelerómetro durante o período de uma semana;
4. privação dos alimentos desaconselhados;
5. tempo gasto com as deslocações e avaliações;
6. os custos associados às deslocações.

Respeito pela liberdade e autonomia do sujeito da investigação

A liberdade e a autonomia estão salvaguardadas na informação prestada ao participante e no seu direito de recusar em qualquer momento a participação neste estudo.

Confidencialidade dos dados

A confidencialidade dos dados é garantida pelo investigador e é transmitida ao participante no texto do convite para participar.

Obtenção do consentimento informado

Os participantes serão informados de todos os procedimentos e fornecerão o consentimento informado para participar no estudo por escrito.

Autorizações necessárias
Estão previstas.

Conflitos de interesse
Este projeto de investigação será financiado pela Farmodiética S.A. e os investigadores declaram não ter conflitos de interesse na condução deste estudo e na divulgação dos achados.

Responsabilidade em caso de ocorrência de danos:
Será assumida pelo investigador principal.

Continuação do tratamento/Seguimento de problemas identificados:
Não referido na documentação entregue. Recomenda-se aos investigadores que revelem os problemas identificados aos participantes e que estes sejam encaminhados para o seu médico assistente.

Política de proteção de dados:
A política de proteção de dados deste projeto é revelada aos potenciais participantes no Termo de Consentimento Informado.
Os dados ficarão sob a responsabilidade da FCNAUP e os documentos em papel serão destruídos cinco anos após o término do estudo.
Está em desenvolvimento um Acordo de Subcontratação para o Tratamento de Dados Pessoais entre a FCNAUP e a Farmodiética S.A., cuja versão intermédia nos foi enviada, em conjunto com a restante documentação.

Curriculum do investigador e equipa de investigação:
São adequados ao estudo em questão.

Conclusão

Pelo exposto, o projeto de investigação mostra-se cientificamente justificado e sem limitações do ponto de vista ético, pelo que recebeu o parecer favorável desta Comissão.

Pedimos que nos seja enviado um relatório final do estudo, após a conclusão do mesmo.

Faculdade de Ciências da Nutrição e Alimentação da Universidade do Porto, 13/9/2021

O Relator	O Presidente
Assinado por : **TERESA MARIA DE SERPA PINTO FREITAS DO AMARAL**<br>Num. de Identificação: 06938005<br>Profª. Doutora Teresa Amaral	Profª. Doutora Sara Rodrigues
