# Supplementary material for: The BREAK study protocol: Effects of intermittent energy restriction on adaptive thermogenesis during weight loss and its maintenance
Source: PLoS One. 2023 Nov 13;18(11):e0294131. doi: 10.1371/journal.pone.0294131 (PMC10642783; doi:10.1371/journal.pone.0294131)
Supplement: S2 File — (PDF) [file pone.0294131.s003.pdf]

ETHICS COMMITTEE

OPINION NO. 31/2021/CEFCNAUP/2021

Project title:

"Effects of intermittent energy restriction on adaptive thermogenesis and successful weight loss maintenance".

Submitted by:

Filipa Maria Teresa Cortez Afonso Faria, student in the Doctoral Program in Clinical Nutrition at the Faculty of Nutrition and Food Sciences of the University of Porto.

The project is part of her doctoral work and is supervised by Prof. Dr. Vítor Hugo Teixeira (Faculty of Nutrition and Food Sciences, University of Porto) and co-supervised by Prof. Dr. Analiza Mónica Silva (Faculty of Human Motricity, University of Lisbon).

Institutions involved in the study:

Faculty of Nutrition and Food Sciences of the University of Porto, Faculty of Human Motricity of the University of Lisbon and private clinic Farmodiética S.A.

Rapporteur:

Teresa Amaral

Objectives of the study

The applicant wants to know whether intermittent energy restriction (two weeks alternating with one week of neutral energy balance), when compared to continuous energy restriction, results in greater weight and fat mass loss, attenuated loss of fat-free mass, less adaptive thermogenesis and a better metabolic profile.

Relevance and design of the study

The study is justified and the methodological aspects are well described. It is proposed to carry out an experimental study with two parallel groups, in which 74 obese adult women aged between 20 and 45 will participate, randomly divided into the study groups. The intervention group will undergo intermittent energy restriction and the control group will undergo continuous energy restriction.

The success of maintaining the lost weight will be assessed at a later stage of neutral energy balance.

This study will be publicized by registering on https://clinicaltrials.gov/, in the media and on social networks.

Benefits/risks

The applicants have clearly identified the possible benefits of this intervention, which are weight loss, optimization of body composition, attenuation of adaptive thermogenesis, improvements in the metabolic profile, well-being and increased self-confidence associated with weight loss and learning and adopting a healthy lifestyle.

Possible risks/discomforts are mentioned:

1. use of a mask during indirect calorimetry;
2. collection of 5 mL of blood for serum levels of free T3 and T4, insulin, leptin and cortisol;
3. using an accelerometer for a period of one week;
4. deprivation of inadvisable foods;
5. time spent traveling and evaluating;
6. the costs associated with travel.

Respect for the freedom and autonomy of the research subject

Freedom and autonomy are safeguarded in the information provided to the participant and in their right to refuse participation in this study at any time.

Data confidentiality

The confidentiality of the data is guaranteed by the researcher and is passed on to the participant in the invitation to take part.

Obtaining informed consent

Participants will be informed of all procedures and will provide written informed consent to participate in the study.

Necessary authorizations
They are planned.

Conflicts of interest
This research project will be funded by Farmodiética S.A. and the researchers declare that they have no conflicts of interest in conducting this study and disseminating the findings.

Liability in the event of damage:
It will be taken over by the principal investigator.

Continuation of treatment/Follow-up of identified problems:
Not mentioned in the documentation provided. It is recommended that the researchers disclose the problems identified to the participants and that they be referred to their treating doctor.

Data protection policy:
The data protection policy of this project is disclosed to potential participants in the Informed Consent Form.
The data will remain the responsibility of FCNAUP and the paper documents will be destroyed five years after the end of the study.
A Subcontracting Agreement for the Processing of Personal Data is being developed between FCNAUP and Farmodiética S.A., an intermediate version of which has been sent to us together with the other documentation.

Curriculum of the researcher and research team:
They are suitable for the study in question.

Conclusion

In view of the above, the research project is scientifically justified and has no ethical limitations, and has therefore received the favorable opinion of this Committee.

Please send us a final report on the study once it has been completed.

Faculty of Nutrition and Food Sciences, University of Porto, 13/9/2021

The	RapporteurThe President
Signed by : **TERESA MARIA DE SERPA PINTO FREITAS DO AMARAL**<br>ID number: 06938005	
Prof. Dr. Teresa Amaral	Prof. Dr. Sara Rodrigues
