# Supplementary material for: The BREAK study protocol: Effects of intermittent energy restriction on adaptive thermogenesis during weight loss and its maintenance
Source: PLoS One. 2023 Nov 13;18(11):e0294131. doi: 10.1371/journal.pone.0294131 (PMC10642783; doi:10.1371/journal.pone.0294131)
Supplement: S3 File — (PDF) [file pone.0294131.s004.pdf]

Resumo do Projeto

Título: Efeitos da restrição energética intermitente na termogénese adaptativa e no sucesso da manutenção do peso perdido

Orientador: Prof. Dr. Vítor Hugo Teixeira

Co-orientadora: Prof. Dra. Analiza Mónica Silva

Doutoranda: Filipa Maria Teresa Cortez Afonso Faria

Pertinência do Estudo

É sabido que um dos principais desafios do tratamento da obesidade consiste na manutenção do peso perdido a longo prazo (Anderson, Konz, Frederich, & Wood, 2001; Barte, Ter Bogt, Bogers, Teixeira, Blissmer, Mori, & Bemelmans, 2010; Curioni & Lourenço, 2005; Tsismenakis, Christophi, Burress, Kinney, Kim, & Kales, 2009; Wadden, Butryn, & Byrne, 2004), devido à elevada taxa de reganho de peso. O insucesso na manutenção do peso perdido prende-se não só com a dificuldade na manutenção de estilos de vida saudáveis a longo prazo, mas também com o facto do organismo reagir à restrição energética (RE) e à redução ponderal com mecanismos adaptativos compensatórios (Muller, Enderle & Bosy-Westphal, 2016), nomeadamente a redução da taxa metabólica de repouso (TMR) num fenómeno denominado de termogénese adaptativa (TA), levando a uma menor eficácia da perda de peso (Byrne, Sainsbury, King, Hills, & Wood, 2018; Muller, Enderle & Bosy-Westphal, 2016).

Apesar da REC ser a abordagem mais comum na intervenção nutricional para a obesidade, acarreta uma série de respostas comportamentais, metabólicas e endócrinas que podem interferir na adesão terapêutica, condicionar negativamente a redução ponderal e predispor o indivíduo para o reganho de peso, quando o processo de RE termina. Por outro lado, a REI parece poder ser uma alternativa viável à REC, com vantagens no âmbito da perda de peso, saciedade e aumento da taxa metabólica de repouso (Peos, Norton, Helms, Galpin, & Fournier, 2018).

Desta forma, considerando esta possível oportunidade terapêutica para a obesidade, este estudo pretende avaliar se uma REI com um padrão de duas semanas em restrição alternada com uma semana de balanço energético neutro, quando comparada com a REC, resulta numa maior perda de peso e massa gorda (MG), atenuação da perda de massa isenta de gordura (MIG), e menor TA.

Metodologia

Esta investigação acontecerá apenas após parecer positivo emitido pela Comissão de Ética da Faculdade de Ciências da Nutrição e Alimentação da Universidade do Porto, e será conduzida de acordo com a declaração de Helsínquia para estudos humanos.

Todos os participantes serão informados sobre os possíveis riscos da investigação antes de darem o seu consentimento escrito referente à sua participação no estudo em questão. A privacidade dos participantes e confidencialidade dos dados será assegurada, durante e após a investigação, de acordo com a legislação vigente.

Desenho do Estudo

Estudo clínico randomizado com 2 grupos paralelos, a ser realizado em mulheres adultas (20-45 anos) com obesidade (Índice de Massa Corporal (IMC) entre 30 e 39,9 kg/m^2). O grupo controlo será o da REC e o grupo em estudo da REI, com uma amostra total de 74 mulheres.

O estudo será dividido em 3 fases, a primeira com a duração de 2 semanas, em que ambos os grupos estarão em balanço energético neutro, a segunda, fase de intervenção com 16 semanas, em que o grupo REC estará em RE durante este período, e o grupo REI alternará 2 semanas de RE com 1 de balanço energético neutro (total de 23 semanas na REI, das quais 16 em RE). A terceira fase consiste em 8 semanas de balanço energético neutro. 12 meses após o término da terceira fase do estudo será avaliado o sucesso da manutenção do peso perdido.

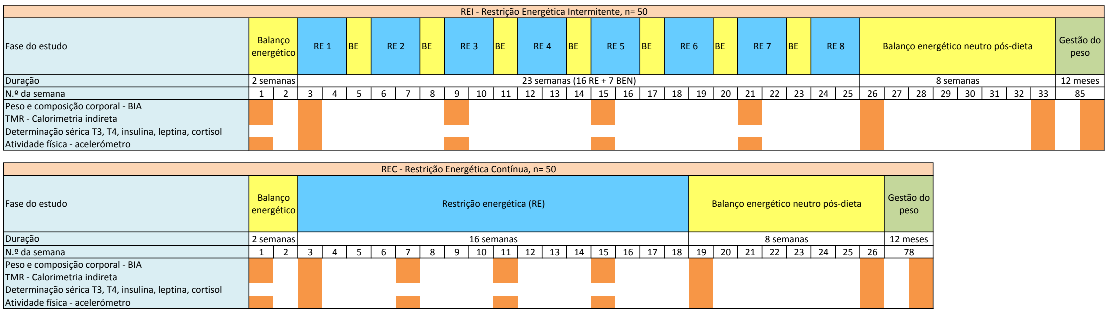

Figura 1 – Descrição esquemática das fases do estudo clínico.

Legenda: BE – balanço energético neutro, RE – restrição energética, BIA – análise de bioimpedância, TMR – taxa metabólica em repouso.

Participantes no estudo

Critérios de inclusão: mulheres obesas, com Índice de Massa Corporal (IMC) entre 30 e 39,9 kg/m^2, peso estável (± 4 kg nos últimos 6 meses), inativas (não cumpre 150 minutos por semana de atividade física de intensidade pelo menos moderada ou 75 minutos por semana de atividade física de intensidade vigorosa, avaliado por acelerometria), idade entre os 20 e 45 anos, disponibilidade para serem randomizadas para qualquer um dos grupos (controlo ou intervenção), que se comprometam a seguir o protocolo a que foram designadas na íntegra, nomeadamente as orientações relativas à intervenção nutricional e de estilo de vida, assim como com a presença e participação em todos momentos de avaliação.

Critérios de exclusão: patologia oncológica, autoimune, renal, hepática (com exceção da esteatose hepática), cardíaca, psiquiátrica, diabetes, patologia inflamatória intestinal crónica, ou outras condições clínicas que afetem a homeostase do balanço energético, menopausa, patologia hormonal ou da tiróide, uso de medicamentos que promovem aumento de peso, nomeadamente corticoesteroides, antidepressivos, ansiolíticos, estabilizadores de humor, antipsicóticos, utilização de medicamentos e/ou suplementos alimentares para perda de peso nos últimos 3 meses, tentativa prévia de emagrecimento no último mês, gravidez e/ou aleitamento há menos de 6 meses, a planear engravidar nos próximos 2 anos, consumo abusivo de álcool auto-reportado ou abuso de substâncias nos últimos 12 meses, consumo atual de mais de 14 bebidas alcoólicas por semana, e/ou em tratamento ou reabilitação para qualquer um destes comportamentos de adição, cirurgia ou internamento hospitalar no último mês, infeção por Covid-19 há menos de 3 meses ou que venha a ser contraída na fase de intervenção do estudo.

Recrutamento e seleção da amostra

Um total de 74 mulheres com os critérios identificados será selecionado. A divulgação do ensaio clínico será feita através do registo no https://clinicaltrials.gov/, nos *media* e redes sociais. A seleção das participantes no estudo será feita em dois momentos: inicialmente via email e/ou telefone, de forma a rastrear possíveis critérios de exclusão, prestar esclarecimentos adicionais, e agendar entrevista presencial; num segundo momento, em entrevista presencial, onde será avaliado o cumprimento dos critérios de inclusão/exclusão, prestada informação detalhada sobre os objetivos do estudo, procedimentos e avaliações, assim como avaliação da motivação para a perda de peso. As entrevistas, consultas e recolha de dados terão lugar em local a definir, com as condições adequadas para a recolha.

Randomização

A seleção das 74 participantes no estudo será efetuada em duas fases (37 participantes x 2) devido ao número de equipamentos disponíveis e pelo facto das avaliações terem de ser efetuadas em jejum (aproximadamente entre as 7h30 e as 10h30). O primeiro grupo de 37 participantes será randomizado para um dos dois braços do estudo, nomeadamente grupo intervenção ou controlo, através de um esquema de randomização automático gerado por computador, que será controlado pelo investigador principal. A randomização terá em consideração a eventual existência de diferenças nas variáveis demográficas e de composição corporal (idade, peso, IMC, massa gorda e MIG). Após a conclusão da randomização será feito o agendamento da primeira visita do estudo, que dará início ao arranque do estudo: fase 1 – balanço energético neutro, e a calendarização das próximas 7 visitas será entregue às participantes.

Determinação das necessidades energéticas e intervenção nutricional durante o balanço energético neutro

O balanço energético neutro consistirá num plano alimentar com 100% das necessidades energéticas de cada participante. Por sua vez, as necessidades energéticas diárias (NED) serão calculadas em função da TMR recolhida via calorimetria indireta e atividade física determinada através dos dados recolhidos por um acelerómetro ActiGraph wGT3X-BT (ActiGraph, Pensacola, Florida, Estados Unidos da América). A distribuição em termos de macronutrientes será a seguinte: 35% proteína, 35% hidratos de carbono e 30% lípidos. Cada indivíduo irá receber um plano alimentar individualizado, de acordo com as NED, e tendo em conta as suas preferências alimentares. Será também fornecido aos participantes uma balança digital, onde deverão pesar-se todos os dias em jejum, sem roupa. Caso registem um aumento do peso consecutivo por 3 dias ou o aumento não consecutivo seja superior a 1Kg, os participantes terão indicações claras de como ajustar o seu plano alimentar, no sentido de reduzir e estabilizar o peso.

Intervenção nutricional na restrição energética

A RE definida em ambos os grupos será de 33% face às necessidades energéticas diárias, calculadas para cada indivíduo (Byrne, Sainsbury, King, Hills, & Wood, 2018; Coutinho, Halset, Gasbakk, Rehfeld, Kulseng, Truby, & Martins, 2018). Considerando que a RE leva à redução da TMR, a mesma será determinada a cada 4 semanas de RE durante a fase de intervenção, de forma a ajustar o plano alimentar, e garantir que se mantêm no mesmo intervalo de RE. Cada participante receberá um plano alimentar individualizado e continuará a monitorizar o seu peso diariamente. A distribuição em termos de macronutrientes será a mesma da fase de balanço energético: 35% proteína, 35% hidratos de carbono e 30% lípidos.

Recolha e tratamento de dados

A recolha de dados antropométricos, composição corporal, e avaliação da TMR será efetuada de manhã, após jejum noturno de pelo menos 10 horas, em oito momentos: *baseline* em balanço energético neutro, início da restrição energética (16 semanas no grupo REC e 16 semanas + 7 no grupo REI), a cada 4 semanas de RE na fase de intervenção (4 semanas de RE com 2 de BE no grupo REI); início e final da fase de balanço energético pós-dieta e após 12 meses da conclusão da intervenção (Byrne, Sainsbury, King, Hills, & Wood, 2018). Os participantes receberão indicação para não tomar diuréticos nos 7 dias anteriores à realização do exame, não ingerir bebidas alcoólicas nem café nas 48 horas anteriores, não realizar atividades físicas extenuantes 24 horas antes e urinar 30 minutos antes dos testes e medições (Khalil, Mohktar, & Ibrahim, 2014).

Antropometria e composição corporal

Todas as medições serão efetuadas sem roupa, utilizando uma bata descartável, de forma a preservar o conforto das participantes, e sem calçado. A avaliação antropométrica deverá ser realizada de acordo com os procedimentos da Orientação Avaliação Antropométrica do Adulto da Direção Geral da Saúde (DGS, 2013). O peso e altura das participantes será avaliado através de uma balança digital Seca com estadiómetro Seca 704 s, com intervalos de 0,1 kg e 0,1 cm (Seca, Hamburgo, Alemanha). O índice de massa corporal será calculado através da fórmula [peso (kg)/altura^2(m^2)].

Análise de Bioimpedância (BIA)

A impedância bioelétrica será avaliada por um analisador de bioimpedância BIA-101 BIVA PRO (Akern srl, Florença, Itália). A percentagem de massa gorda (MG) e massa isenta de gordura (MIG) será obtida utilizando o software Bodygram® (AkernSrl., Florença, Italia). (Toselli, Badicu, Bragonzoni, Spiga, Mazzuca, & Campa, 2020). Antes do teste, será indicado aos indivíduos para se deitarem na posição decúbito dorsal, com os braços e pernas abduzidos num ângulo de 45 graus durante 10 minutos (Silva, Nunes, Matias, Jesus, Francisco, Cardoso, ... Minderico, 2019).

Taxa metabólica em repouso

A determinação da TMR será efetuada através de calorimetria indireta (Haugen, Chan, Li, 2007) com recurso ao aparelho COSMED Fitmate (Cosmed, Roma, Itália) (Lee, Bassett, Thompson, & Fitzhugh, 2011). O teste deverá ser efetuado entre as 7h30 e 10h30 da manhã, após um jejum noturno de pelo menos 10h. Os participantes serão aconselhados a reduzir a sua atividade física ao mínimo no dia do teste, até que o mesmo tenha tido início. O teste deverá acontecer num ambiente termicamente neutro (22º C), em supino e numa posição confortável. Durante o teste, as participantes deverão manter-se o mais relaxadas possível, sem adormecer, sem falar e mantendo-se imóveis. O teste terá a duração de 30 minutos, descartando-se os primeiros 10 minutos. O aparelho de calorimetria será conectado à máscara e a TMR será calculada utilizando os valores médios mais baixos de VO2 e VCO2 medidos durante 10 minutos consecutivos, considerando um coeficiente de variação inferior a 5% nesse intervalo (Byrne, Sainsbury, King, Hills, & Wood, 2018).

Termogénese Adaptativa

Para calcular a TMR esperada no *baseline*, será criada uma equação de regressão utilizando a MIG e MG como os preditores independentes. Esta equação será depois utilizada para predizer a TMR em cada fase do estudo, utilizando os valores de MIG e MG nestes momentos. Para considerar as eventuais adaptações na TMR não previstas pela alteração da MIG, a resposta

adaptativa será calculada da seguinte forma: 1 menos o rácio entre a TMR atual e a TMR esperada. Valores positivos indicarão um decréscimo na TMR para além do previsto devido a alterações na composição corporal (TMR atual inferior à esperada), assim como resultados negativos indicarão o inverso (Thomas, Bouchard, Church, Slentz, Kraus, Redman, ... Heymsfield, 2012).

Atividade física e dispêndio energético total

A atividade física será determinada através do acelerómetro ActiGraph wGT3X-BT (ActiGraph, Pensacola, Estados Unidos da América), que irá expressar os minutos por dia despendidos em diferentes atividades. A ativação dos aparelhos, download e processamento será feito com recurso ao software Actilife (v.6.9.1).

Os valores de ponto de corte utilizados para definir a intensidade da atividade física e o tempo médio despendido de acordo com a intensidade (sedentária, ligeira, moderada ou vigorosa) serão os seguintes: sedentária < 100 contagens por minuto^{-1}; ligeira: 100-2019 contagens por minuto^{-1}; moderada: 2020-5998 contagens por minuto^{-1} (correspondente a 3-5.9 METs); vigorosa: ≥ 5999 contagens por minuto^{-1} (correspondente to ≥6 METs). Para que a determinação da atividade física seja possível será necessário que os indivíduos utilizem o acelerómetro durante pelo menos três dias válidos. Um dia válido corresponde a uma utilização durante 600 ou mais minutos (10 ou mais horas), sendo que períodos de pelo menos 60 minutos consecutivos sem qualquer intensidade será considerado tempo de não utilização (Silva, Nunes, Matias, Jesus, Francisco, Cardoso, ... Minderico, 2019).

O dispêndio energético total (DET) será calculado utilizando as equações de Crouter e colegas. O dispêndio energético relacionado com a atividade física será calculado da seguinte forma: DET menos (0,1 x DET + TMR), assumindo que o efeito térmico dos alimentos representa 10% do DET (Crouter, Kuffel, Haas, Frongillo, & Bassett, 2010; Troiano, Berrigan, Dodd, Mâsse, Tilert, & McDowell, 2008; Ward, Evenson, Vaughn, Rodgers, & Troiano, 2005).

Determinação hormonais séricas

A recolha de sangue (5 mL) para a determinação sérica de T3 e T4 livre, insulina, leptina e cortisol será efetuada em jejum. Estas recolhas serão efetuadas em quatro momentos: início da intervenção de restrição energética, início e final do balanço energético pós-dieta e 12 meses após final da intervenção.

Tratamento de dados

O tratamento dos dados será realizado através do software estatístico SPSS versão 27.0, 2020 (SPSS Inc., an IBM Company, Chicago IL, USA) com nível de significância de 0,05. Estatísticas descritivas serão calculadas (média, desvio padrão e intervalo) em cada momento da recolha de dados. As diferenças entre condições nos oito momentos de avaliação serão analisadas utilizando medidas repetidas (para comparações não ajustadas) e correção de Bonferroni para comparações ajustadas. Medidas repetidas também serão usadas para testar se ocorrem mudanças comportamentais compensatórias. Para determinar a TA serão utilizadas análises de regressão linear (variável dependente: TMR e variáveis independentes: MIG e MG).

Tamanho da amostra

Considerando um erro tipo I de 5% e uma potência de 95% para detetar diferenças nas variáveis dependentes, com significado estatístico e um tamanho do efeito grande (0,9339003) serão necessários um total de 26 participantes por grupo (software GPower versão 3.1.9.6).

Assumindo uma taxa de desistência de 30% ao longo do estudo (Byrne, Sainsbury, King, Hills, & Wood, 2018), serão recrutados 74 participantes (37 em cada grupo).

Divulgação da investigação à comunidade científica e participantes

Os resultados obtidos com o estudo serão divulgados à comunidade científica através da submissão de artigos científicos com revisão por pares e presença em conferências/congressos nacionais e/ou internacionais, assim como aos participantes mediante reuniões de grupo e/ou envio de email com resultados principais.

Avaliação benefício-risco

Os benefícios do estudo ultrapassam em larga escala os eventuais riscos. Os benefícios identificados são: i) perda de peso e otimização da composição corporal; ii) melhorias metabólicas decorrentes da perda de peso clinicamente significativa; iii) eventual atenuação da termogénese adaptativa, facilitando a perda e manutenção do peso perdido; iv) bem-estar e aumento da auto-confiança associadas à perda de peso; v) adoção de um estilo de vida saudável, com aprendizagem de estratégias nutricionais e aquisição de ferramentas, com vista à adoção de hábitos alimentares saudáveis e gestão eficaz do peso corporal. Os eventuais riscos incluem: i) desconforto com utilização da máscara durante a avaliação de calorimetria indireta; ii) desconforto com a recolha de sangue para as determinações séricas de T3 e T4 livre, insulina, leptina e cortisol; iii) utilização de um acelerómetro colocado na zona da anca durante o período de uma semana; iv) privação de alimentos de elevada densidade energética ou de qualidade nutricional insuficiente, que podem constituir uma fonte de prazer imediato para os participantes; v) tempo despendido com as visitas ao laboratório/clínica, bem como os custos associados.

Conflitos de interesse

Os investigadores declaram não ter conflitos de interesse para a condução deste estudo.

Fonte de financiamento

Este projeto de investigação é financiado pela Farmodiética S.A., sem que isso tenha influência na normal condução do estudo, nem sobre os resultados apurados e divulgação dos mesmos.

Política de proteção de dados

A informação recolhida durante o estudo será utilizada apenas pela equipa de investigação, estando garantido o anonimato dos participantes e a confidencialidade dos dados. Os dados pessoais não estarão em discos, nem é permita a utilização de redes públicas, sendo apenas utilizadas ligações seguras encriptadas como ligações por VPN. Os documentos em papel serão guardados em sala fechada.

Os dados estão sob a responsabilidade da FCNAUP e serão utilizados apenas para efeitos de defesa de tese de doutoramento. Os documentos em suporte de papel serão destruídos após a construção da matriz de tratamento dos dados. Está previsto um Acordo de Subcontratação para o Tratamento de Dados Pessoais entre a FCNAUP e a Farmodiética S.A., em anexo.

A política de proteção de dados deste projeto de investigação está disponível no Anexo I do Termo de Consentimento Informado.

Referências Bibliográficas

Anderson, J.W., Konz, E.C., Frederich, R.C., & Wood, C.L. (2001). Long-term weight-loss maintenance: a meta-analysis of US studies. ***American Journal of Clinical Nutrition, 74***(5), 579-84. doi 10.1093/ajcn/74.5.579

Barte, J.C.M., Ter Bogt, N.C.W., Bogers, R.P., Teixeira, P.J., Blissmer, B., Mori, T.A. & Bemelmans, W.J.E. (2010). Maintenance of weight-loss after lifestyle interventions for overweight and obesity, a systematic review. ***Obesity Reviews, 11***(12):899-906. doi 10.1111/j.1467-789X.2010.00740.x

Byrne, N.M., SainsburyA., King N.A., Hills, A.P., & Wood, R.E. (2018). Intermittent energy restriction improves weight loss efficiency in obese men: the MATADOR study. ***International Journal of Obesity, 42***, 129-138. doi 10.1038/ijo.2017.206

Coutinho, S.R., Halset, E.H., Gasbakk, S., Rehfeld, J.F., Kulseng, B., Truby, H., & Martins, C. (2018). Compensatory mechanisms activated with intermittent energy restriction: a randomized control trial. ***Clinical Nutrition, 37***, 815-823.

Crouter, S.E., Kuffel, E., Haas, J.D., Frongillo, E.A., & Bassett, D.R. Jr. (2010). Refined two-regression model for the ActiGraph accelerometer. **Medicine and Science in Sports and Exercise, 42**(5), 1029-37. doi: 10.1249/MSS.0b013e3181c37458

Curioni, C.C., & Lourenço, P.M. (2005). Long-term weight loss after diet and exercise: a systematic review. ***International Journal of Obesity, 29***(10), 1168-74. doi 10.1038/sj.ijo.0803015

Haugen, H.A., Chan, L.N., Li, F. (2007). Indirect calorimetry: a pratical guide for clinicians. ***Nutrition in Clinical Practice, 22***(4), 377-388. doi 10.1177/0115426507022004377

Khalil, S.F., Mohktar, M.S., & Ibrahim, F. (2014). The theory and fundamentals of bioimpedance analysis in clinical status monitoring and diagnosis of diseases. ***Sensors, 14***, 10895-10928. doi 10.3390/s140610895

Lee, J.M., Bassett, D.R. Jr., Thompson, D.L., Fitzhugh, E.C. (2011). Validation of the Cosmed Fitmate for prediction of maximal oxygen consumption. ***The Journal of Strength and Conditioning Research, 25***(9):2573-9. doi: 10.1519/JSC.0b013e3181fc5c48

Muller, M.J., Enderle, J., & Bosy-Westphal, A. (2016). Changes in energy expenditure with weight gain and weight loss in humans. ***Current Obesity Reviews, 5***, 413-423. doi 10.1007/s1379-016-0237-4

Peos, J.J., Norton, L.E., Helms, E.R., Galpin, A.J., & Fournier, P. (2018). Intermittent dieting: theoretical considerations for the athlete. ***Sports, 7***(1), 22. doi:10.3390/sports7010022

Silva, A.M., Nunes, C.L., Matias, C.N., Jesus, F., Francisco, R., Cardoso, M. ... Minderico, C. (2019). Champ4life Study Protocol: a one-year randomized controlled trial of a lifestyle intervention for inactive former elite athletes with overweight/obesity. ***Nutrients, 12***, 286. doi:10.3390/nu12020286

Thomas, D.M., Bouchard, C., Church. T., Slentz, C., Kraus, WE, Redman LM, ... Heymsfield, S.B. (2012). Why do individuals not lose more weight from an exercise intervention at a defined dose? An energy balance analysis. ***Obesity reviews, 13***(10),835-47. doi:10.1111/j.1467-789X.2012.01012.x

Toselli, S., Badicu, G., Bragonzoni, L., Spiga, F., Mazzuca, P., & Campa, F. (2020). Comparison of the effect of different resistance training frequencies on phase angle and handgrip strength in obese women: a randomized controlled trial. International ***Journal of Environmental Research and Public Health***, 17, 1163. doi:10.3390/ijerph17041163

Troiano, R.P., Berrigan, D., Dodd, K.W., Mâsse, L.C., Tilert, T., & McDowell, M. (2008). Physical activity in the United States measured by accelerometer. **Medicine and Science in Sports and Exercise, 40**(1), 181-8.

Tsismenakis, A.J., Christophi, C.A., Burress, J.W., Kinney, A.M., Kim, M., & Kales, S.N. (2009). The obesity epidemic and future emergency responders. ***Obesity, 17***(8), 1648-50. doi: 10.1038/oby.2009.63

Wadden, T.A., Butryn, M.L., & Byrne, K.J. (2004). Efficacy of lifestyle modification for long-term weight control. ***Obesity Research, 12***, Suppl,151S-62S. doi 10.1038/oby.2004.282

Ward, D.S., Evenson, K.R., Vaughn, A., Rodgers, A.B., & Troiano, R.P. (2005). Accelerometer use in physical activity: best practices and research recommendations. **Medicine and Science in Sports and Exercise, 37**(11 Suppl), S582-8.
