# Supplementary material for: The BREAK study protocol: Effects of intermittent energy restriction on adaptive thermogenesis during weight loss and its maintenance
Source: PLoS One. 2023 Nov 13;18(11):e0294131. doi: 10.1371/journal.pone.0294131 (PMC10642783; doi:10.1371/journal.pone.0294131)
Supplement: S4 File — (PDF) [file pone.0294131.s005.pdf]

Project Summary

Title: Effects of intermittent energy restriction on adaptive thermogenesis and successful weight loss maintenance

Advisor: Prof. Dr. Vítor Hugo Teixeira

Co-advisor: Prof. Dr. Analiza Mónica Silva

Doctoral student: Filipa Maria Teresa Cortez Afonso Faria

Relevance of the Study

It is well known that one of the main challenges in treating obesity is maintaining the weight lost in the long term (Anderson, Konz, Frederich, & Wood, 2001; Barte, Ter Bogt, Bogers, Teixeira, Blissmer, Mori, & Bemelmans, 2010; Curioni & Lourenço, 2005; Tsismenakis, Christophi, Burress, Kinney, Kim, & Kales, 2009; Wadden, Butryn, & Byrne, 2004), due to the high rate of weight regain. The failure to maintain the weight lost is related not only to the difficulty in maintaining healthy lifestyles in the long term, but also to the fact that the body reacts to energy restriction (ER) and weight reduction with compensatory adaptive mechanisms (Muller, Enderle & Bosy-Westphal, 2016), namely the reduction in resting metabolic rate (RMR) in a phenomenon called adaptive thermogenesis (AT), leading to less effective weight loss (Byrne, Sainsbury, King, Hills, & Wood, 2018; Muller, Enderle & Bosy-Westphal, 2016).

Although CR is the most common approach in nutritional intervention for obesity, it entails a series of behavioral, metabolic and endocrine responses that can interfere with therapeutic adherence, negatively condition weight reduction and predispose the individual to weight regain when the CR process ends. On the other hand, REI seems to be a viable alternative to CER, with advantages in terms of weight loss, satiety and increased resting metabolic rate (Peos, Norton, Helms, Galpin, & Fournier, 2018).

Thus, considering this possible therapeutic opportunity for obesity, this study aims to assess whether an IER with a pattern of two weeks of restriction alternated with one week of neutral energy balance, when compared to the REC, results in greater weight and fat mass (WM) loss, attenuation of the loss of fat-free mass (FFM), and lower AT.

Methodology

This research will only take place after a positive opinion has been issued by the Ethics Committee of the Faculty of Nutrition and Food Sciences of the University of Porto, and will be conducted in accordance with the Helsinki declaration for human studies.

All participants will be informed of the possible risks of the research before giving their written consent to take part in the study in question. Participants' privacy and data confidentiality will be ensured during and after the research, in accordance with current legislation.

Study design

Randomized clinical study with 2 parallel groups, to be carried out on adult women (20-45 years old) with obesity (Body Mass Index (BMI) between 30 and 39.9 kg/m^2). The control group will be the REC and the study group the REI, with a total sample of 74 women.

The study will be divided into 3 phases, the first lasting 2 weeks, in which both groups will be in neutral energy balance, the second, 16-week intervention phase, in which the REC group will be in RE during this period, and the REI group will alternate 2 weeks of RE with 1 of neutral energy balance (total of 23 weeks in REI, of which 16 in RE). The third phase consists of 8 weeks of neutral energy balance. 12 months after the end of the third phase of the study, the success of maintaining the lost weight will be evaluated.

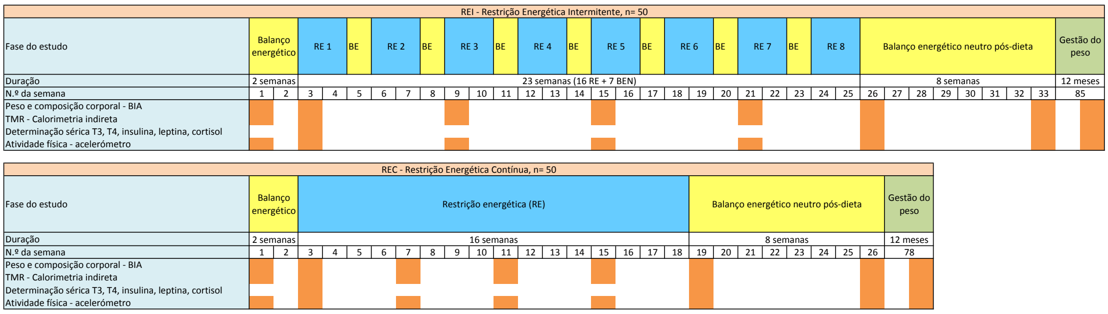

Figure 1 - Schematic description of the clinical study phases.

Legend: BE - neutral energy balance, RE - energy restriction, BIA - bioimpedance analysis, TMR - resting metabolic rate.

Participants in the study

Inclusion criteria: obese women, with a Body Mass Index (BMI) between 30 and 39.9 kg/m^2 , stable weight (± 4 kg in the last 6 months), inactive (not completing 150 minutes per week of at least moderate intensity physical activity or 75 minutes per week of vigorous intensity physical activity, assessed by accelerometry), aged between 20 and 45 years, willingness to be randomized to any of the groups (control or intervention), who commit to following the protocol to which they have been assigned in full, namely the guidelines for nutritional and lifestyle intervention, as well as attendance and participation at all assessment moments.

Exclusion criteria: Oncological, autoimmune, renal, hepatic (with the exception of hepatic steatosis), cardiac, psychiatric, diabetes, chronic inflammatory bowel disease, or other clinical conditions affecting energy balance homeostasis, menopause, hormonal or thyroid pathology, use of medications that promote weight gain, namely corticosteroids, antidepressants, anxiolytics, mood stabilizers, antipsychotics, use of medications and/or food supplements for weight loss in the last 3 months, previous attempt to lose weight in the last month, pregnancy and/or breastfeeding less than 6 months ago, planning to become pregnant in the next 2 years, self-reported alcohol abuse or substance abuse in the last 12 months, current consumption of more than 14 alcoholic drinks per week, and/or in treatment or rehabilitation for any of these addictive behaviors, surgery or hospitalization in the last month, Covid-19 infection less than 3 months ago or that will be contracted in the intervention phase of the study.

Recruitment and selection of the sample

A total of 74 women with the identified criteria will be selected. The clinical trial will be publicized by registering on https://clinicaltrials.gov/, the *media* and social networks.

Participants in the study will be selected in two stages: initially via email and/or telephone, in order to screen for possible exclusion criteria, provide additional clarification and schedule a face-to-face interview; secondly, in a face-to-face interview, where compliance with the inclusion/exclusion criteria will be assessed, detailed information on the study's objectives, procedures and assessments will be provided, as well as an assessment of motivation for weight loss. The interviews, consultations and data collection will take place in a location to be defined, with the appropriate conditions for collection.

Randomization

The 74 participants in the study will be selected in two phases (37 participants x 2) due to the number of devices available and the fact that the assessments will have to be carried out while fasting (approximately between 7.30am and 10.30am). The first group of 37 participants will be randomized to one of the two arms of the study, namely the intervention or control group, using a computer-generated automatic randomization scheme, which will be controlled by the principal investigator. Randomization will take into account any differences in demographic and body composition variables (age, weight, BMI, fat mass and MIG). Once randomization has been completed, the first study visit will be scheduled, which will initiate the start of the study: phase 1 - neutral energy balance, and the schedule of the next 7 visits will be given to the participants.

Determining energy needs and nutritional intervention during neutral energy balance

The neutral energy balance will consist of a diet plan with 100% of each participant's energy needs. In turn, the daily energy requirements (DER) will be calculated based on the BMR collected via indirect calorimetry and physical activity determined using the data collected by an ActiGraph wGT3X-BT accelerometer (ActiGraph, Pensacola, Florida, United States of America). The macronutrient distribution will be as follows: 35% protein, 35% carbohydrates and 30% lipids. Each individual will receive an individualized diet plan, according to the NEDs and taking into account their dietary preferences. Participants will also be given a digital scale, where they will have to weigh themselves every day on an empty stomach. If there is a consecutive increase in weight for 3 days or if the non-consecutive increase is more than 1 kg, participants will be given clear instructions on how to adjust their diet plan in order to reduce and stabilize their weight.

Nutritional intervention in energy restriction

The RE set for both groups will be 33% of the daily energy needs calculated for each individual (Byrne, Sainsbury, King, Hills, & Wood, 2018; Coutinho, Halset, Gasbakk, Rehfeld, Kulseng, Truby, & Martins, 2018). Considering that CR leads to a reduction in RMT, this will be determined every 4 weeks of CR during the intervention phase, in order to adjust the diet plan and ensure that they remain in the same CR range. Each participant will receive an individualized diet plan and will continue to monitor their weight on a daily basis. The macronutrient distribution will be the same as in the energy balance phase: 35% protein, 35% carbohydrates and 30% lipids.

Data collection and processing

Anthropometric data, body composition and RMR will be collected in the morning, after an overnight fast of at least 10 hours, at eight points in time: *baseline* in neutral energy balance, start of energy restriction (16 weeks in the REC group and 16 weeks + 7 in the REI group), every 4 weeks of RE in the intervention phase (4 weeks of RE with 2 of BE in the REI group); start and end of the post-diet energy balance phase and after 12 months from the conclusion of the

intervention (Byrne, Sainsbury, King, Hills, & Wood, 2018). Participants will be instructed not to take diuretics in the 7 days prior to the test, not to drink alcohol or coffee in the 48 hours prior, not to perform strenuous physical activity 24 hours before and to urinate 30 minutes before the tests and measurements (Khalil, Mohktar, & Ibrahim, 2014).

Anthropometry and body composition

All measurements will be taken without clothing, using a disposable gown in order to preserve the comfort of the participants, and without shoes. The anthropometric assessment will be carried out in accordance with the procedures of the Anthropometric Assessment of Adults Guideline of the Directorate-General for Health (DGS, 2013). The participants' weight and height will be assessed using a Seca digital scale with a Seca 704 s stadiometer, at intervals of 0.1 kg and 0.1 cm (Seca, Hamburg, Germany). Body mass index will be calculated using the formula [weight (kg)/height^2 (m^2)].

Bioimpedance Analysis (BIA)

Bioelectrical impedance will be assessed using a BIA-101 BIVA PRO bioimpedance analyzer (Akern srl, Florence, Italy). The percentage of fat mass (FFM) and fat-free mass (FFM) will be obtained using Bodygram® software (Akern srl, Florence, Italy) (Toselli, Badicu, Bragonzoni, Spiga, Mazzuca, & Campa, 2020). Before the test, individuals will be instructed to lie down in the supine position with their arms and legs abducted at a 45-degree angle for 10 minutes (Silva, Nunes, Matias, Jesus, Francisco, Cardoso, ... Minderico, 2019).

Resting metabolic rate

RMT will be determined using indirect calorimetry (Haugen, Chan, Li, 2007) using the COSMED Fitmate device (Cosmed, Rome, Italy) (Lee, Bassett, Thompson, & Fitzhugh, 2011). The test should be carried out between 7:30 and 10:30 in the morning, after an overnight fast of at least 10 hours. Participants will be advised to reduce their physical activity to a minimum on the day of the test, until it has begun. The test should take place in a thermoneutral environment (22º C), supine and in a comfortable position. During the test, the participants should remain as relaxed as possible, without falling asleep, without talking and remaining still. The test will last 30 minutes, discarding the first 10 minutes. The calorimetry device will be connected to the mask and the RMT will be calculated using the lowest average VO2 and VCO2 values measured during 10 consecutive minutes, considering a coefficient of variation of less than 5% in that interval (Byrne, Sainsbury, King, Hills, & Wood, 2018).

Adaptive Thermogenesis

To calculate the expected TMR at *baseline*, a regression equation will be created using MIG and MG as independent predictors. This equation will then be used to predict the TMR at each stage of the study, using the MIG and MG values at these times. To take into account any adaptations in TMR not predicted by the change in MIG, the adaptive response will be calculated as follows: 1 minus the ratio between the current TMR and the expected TMR. Positive values will indicate a decrease in RMR beyond that predicted due to changes in body composition (current RMR lower than expected), while negative results will indicate the opposite (Thomas, Bouchard, Church, Slentz, Kraus, Redman, ... Heymsfield, 2012).

Physical activity and total energy expenditure

Physical activity will be determined using the ActiGraph wGT3X-BT accelerometer (ActiGraph, Pensacola, USA), which will express the minutes per day spent on different activities. The devices will be activated, downloaded and processed using Actilife software (v.6.9.1).

The cut-off values used to define the intensity of physical activity and the average time spent according to intensity (sedentary, light, moderate or vigorous) will be as follows: sedentary < 100 counts per minute^{-1} ; light: 100-2019 counts per minute^{-1} ; moderate: 2020-5998 counts per minute^{-1} (corresponding to 3-5.9 METs); vigorous: ≥ 5999 counts per minute^{-1} (corresponding to ≥6 METs). In order to determine physical activity, individuals must use the accelerometer for at least three valid days. A valid day corresponds to use for 600 or more minutes (10 or more hours), and periods of at least 60 consecutive minutes without any intensity will be considered non-use time (Silva, Nunes, Matias, Jesus, Francisco, Cardoso, ... Minderico, 2019).

Total energy expenditure (TEE) will be calculated using the equations of Crouter and colleagues. Energy expenditure related to physical activity will be calculated as follows: DET minus (0.1 x DET + TMR), assuming that the thermic effect of food represents 10% of DET (Crouter, Kuffel, Haas, Frongillo, & Bassett, 2010; Troiano, Berrigan, Dodd, Mâsse, Tilert, & McDowell, 2008; Ward, Evenson, Vaughn, Rodgers, & Troiano, 2005).

Determination of serum hormones

Blood samples (5 mL) for serum determination of free T3 and T4, insulin, leptin and cortisol will be taken during fasting. These samples will be taken at four points in time: the beginning of the energy restriction intervention, the beginning and end of the post-diet energy balance and 12 months after the end of the intervention.

Data processing

The data will be processed using SPSS statistical software version 27.0, 2020 (SPSS Inc., an IBM Company, Chicago IL, USA) with a significance level of 0.05. Descriptive statistics will be calculated (mean, standard deviation and range) at each moment of data collection. Differences between conditions at the eight assessment moments will be analyzed using repeated measures (for unadjusted comparisons) and Bonferroni correction for adjusted comparisons. Repeated measures will also be used to test whether compensatory behavioral changes occur. Linear regression analyses will be used to determine AT (dependent variable: TMR and independent variables: MIG and MG).

Sample size

Considering a type I error of 5% and a power of 95% to detect differences in the dependent variables, with statistical significance and a large effect size (0.9339003), a total of 26 participants per group will be needed (GPower software version 3.1.9.6). Assuming a dropout rate of 30% throughout the study (Byrne, Sainsbury, King, Hills, & Wood, 2018), 74 participants will be recruited (37 in each group).

Dissemination of research to the scientific community and participants

The results obtained from the study will be disseminated to the scientific community through the submission of peer-reviewed scientific articles and attendance at national and/or international conferences/congresses, as well as to the participants through group meetings and/or sending emails with the main results.

Benefit-risk assessment

The benefits of the study far outweigh the possible risks. The benefits identified are: i) weight loss and optimization of body composition; ii) metabolic improvements resulting from clinically significant weight loss; iii) possible attenuation of adaptive thermogenesis, facilitating the loss and maintenance of lost weight; iv) well-being and increased self-confidence associated with weight loss; v) adoption of a healthy lifestyle, with learning of nutritional strategies and acquisition of tools, with a view to adopting healthy eating habits and effective management of body weight. Possible risks include: i) discomfort with wearing the mask during the indirect calorimetry assessment; ii) discomfort with blood sampling for serum determinations of free T3 and T4, insulin, leptin and cortisol; iii) use of an accelerometer placed on the hip area for a period of one week; iv) deprivation of energy-dense foods or foods of insufficient nutritional quality, which can be a source of immediate pleasure for the participants; v) time spent visiting the laboratory/clinic, as well as the associated costs.

Conflicts of interest

The researchers declare that they have no conflicts of interest in conducting this study.

Source of funding

This research project is funded by Farmodiética S.A., without this having any influence on the normal conduct of the study, nor on the results obtained and their dissemination.

Data protection policy

The information collected during the study will only be used by the research team, and the anonymity of the participants and the confidentiality of the data will be guaranteed. Personal data will not be on disks, nor will the use of public networks be allowed, only secure encrypted connections such as VPN connections will be used. Paper documents will be kept in a locked room.

The data is the responsibility of FCNAUP and will only be used for the purposes of defending the doctoral thesis. Paper documents will be destroyed once the data processing matrix has been constructed. A Subcontracting Agreement for the Processing of Personal Data between FCNAUP and Farmodiética S.A. is attached.

The data protection policy of this research project is available in Annex I of the Informed Consent Form.

Bibliographical references

Anderson, J.W., Konz, E.C., Frederich, R.C., & Wood, C.L. (2001). Long-term weight-loss maintenance: a meta-analysis of US studies. ***American Journal of Clinical Nutrition, 74***(5), 579-84. doi 10.1093/ajcn/74.5.579

Barte, J.C.M., Ter Bogt, N.C.W., Bogers, R.P., Teixeira, P.J., Blissmer, B., Mori, T.A. & Bemelmans, W.J.E. (2010). Maintenance of weight-loss after lifestyle interventions for overweight and obesity, a systematic review. ***Obesity Reviews, 11***(12):899-906. doi 10.1111/j.1467-789X.2010.00740.x

Byrne, N.M., SainsburyA., King N.A., Hills, A.P., & Wood, R.E. (2018). Intermittent energy restriction improves weight loss efficiency in obese men: the MATADOR study. ***International Journal of Obesity, 42***, 129-138. doi 10.1038/ijo.2017.206

Coutinho, S.R., Halset, E.H., Gasbakk, S., Rehfeld, J.F., Kulseng, B., Truby, H., & Martins, C. (2018). Compensatory mechanisms activated with intermittent energy restriction: a randomized control trial. ***Clinical Nutrition, 37***, 815-823.

Crouter, S.E., Kuffel, E., Haas, J.D., Frongillo, E.A., & Bassett, D.R. Jr. (2010). Refined two-regression model for the ActiGraph accelerometer. **Medicine and Science in Sports and Exercise, 42**(5), 1029-37. doi: 10.1249/MSS.0b013e3181c37458

Curioni, C.C., & Lourenço, P.M. (2005). Long-term weight loss after diet and exercise: a systematic review. ***International Journal of Obesity, 29***(10), 1168-74. doi 10.1038/sj.ijo.0803015

Haugen, H.A., Chan, L.N., Li, F. (2007). Indirect calorimetry: a practical guide for clinicians. ***Nutrition in Clinical Practice, 22***(4), 377-388. doi 10.1177/0115426507022004377

Khalil, S.F., Mohktar, M.S., & Ibrahim, F. (2014). The theory and fundamentals of bioimpedance analysis in clinical status monitoring and diagnosis of diseases. ***Sensors, 14***, 10895-10928. doi 10.3390/s140610895

Lee, J.M., Bassett, D.R. Jr., Thompson, D.L., Fitzhugh, E.C. (2011). Validation of the Cosmed Fitmate for prediction of maximal oxygen consumption. ***The Journal of Strength and Conditioning Research, 25***(9):2573-9. doi: 10.1519/JSC.0b013e3181fc5c48

Muller, M.J., Enderle, J., & Bosy-Westphal, A. (2016). Changes in energy expenditure with weight gain and weight loss in humans. ***Current Obesity Reviews, 5***, 413-423. doi 10.1007/s1379-016-0237-4

Peos, J.J., Norton, L.E., Helms, E.R., Galpin, A.J., & Fournier, P. (2018). Intermittent dieting: theoretical considerations for the athlete. ***Sports, 7***(1), 22. doi:10.3390/sports7010022

Silva, A.M., Nunes, C.L., Matias, C.N., Jesus, F., Francisco, R., Cardoso, M. ... Minderico, C. (2019). Champ4life Study Protocol: a one-year randomized controlled trial of a lifestyle intervention for inactive former elite athletes with overweight/obesity. ***Nutrients, 12***, 286. doi:10.3390/nu12020286

Thomas, D.M., Bouchard, C., Church. T., Slentz, C., Kraus, WE, Redman LM, ... Heymsfield, S.B. (2012). Why do individuals not lose more weight from an exercise intervention at a defined dose?

An energy balance analysis. ***Obesity reviews, 13***(10),835-47. doi:10.1111/j.1467-789X.2012.01012.x

Toselli, S., Badicu, G., Bragonzoni, L., Spiga, F., Mazzuca, P., & Campa, F. (2020). Comparison of the effect of different resistance training frequencies on phase angle and handgrip strength in obese women: a randomized controlled trial. International ***Journal of Environmental Research and Public Health***, 17, 1163. doi:10.3390/ijerph17041163

Troiano, R.P., Berrigan, D., Dodd, K.W., Mâsse, L.C., Tilert, T., & McDowell, M. (2008). Physical activity in the United States measured by accelerometer. **Medicine and Science in Sports and Exercise, 40**(1), 181-8.

Tsismenakis, A.J., Christophi, C.A., Burress, J.W., Kinney, A.M., Kim, M., & Kales, S.N. (2009). The obesity epidemic and future emergency responders. ***Obesity, 17***(8), 1648-50. doi: 10.1038/oby.2009.63

Wadden, T.A., Butryn, M.L., & Byrne, K.J. (2004). Efficacy of lifestyle modification for long-term weight control. ***Obesity Research, 12***, Suppl,151S-62S. doi 10.1038/oby.2004.282

Ward, D.S., Evenson, K.R., Vaughn, A., Rodgers, A.B., & Troiano, R.P. (2005). Accelerometer use in physical activity: best practices and research recommendations. **Medicine and Science in Sports and Exercise, 37**(11 Suppl), S582-8.
