# Supplementary material for: The BREAK study protocol: Effects of intermittent energy restriction on adaptive thermogenesis during weight loss and its maintenance
Source: PLoS One. 2023 Nov 13;18(11):e0294131. doi: 10.1371/journal.pone.0294131 (PMC10642783; doi:10.1371/journal.pone.0294131)
Supplement: S1 Appendix — (PDF) [file pone.0294131.s006.pdf]

CONSENTIMENTO INFORMADO, LIVRE E ESCLARECIDO PARA INVESTIGAÇÃO CIENTÍFICA COM SERES HUMANOS

Título do estudo: Efeitos da restrição energética intermitente na termogénese adaptativa e no sucesso da manutenção do peso perdido

Responsáveis pelo projeto: Prof. Dr. Vítor Hugo Teixeira, Prof. Dra. Analiza Mónica Silva.

O presente documento, designado **Consentimento Informado, Livre e Esclarecido**, possui informação relevante no que se refere ao estudo clínico para o qual foi convidada a participar, assim como aquilo que deve esperar caso decida participar no mesmo. Pedimos assim que leia com atenção toda a informação aqui contida, sendo que deve sentir-se totalmente livre para colocar questões, bem como para discutir a sua participação com terceiros (familiares, amigos ou conhecidos), a fim de decidir a sua participação neste estudo.

Informação geral

O estudo de investigação para o qual está a ser convidada a participar pretende analisar os efeitos de uma determinada intervenção nutricional nas adaptações metabólicas que ocorrem durante o processo de perda de peso, bem como no sucesso da manutenção do peso perdido. Considerando que cumpre com os critérios de elegibilidade, disponibiliza-se através deste documento a integrar o estudo clínico em questão, sendo importante que esteja presente nos vários momentos de avaliação, seguindo o mais possível as orientações nutricionais fornecidas em consulta com vista à perda de peso durante a intervenção, bem como à manutenção do peso perdido após o término da fase de redução ponderal, durante o período de 12 meses.

Após a sua confirmação de intenção de participação, através da assinatura deste documento, será alocada de forma aleatória a um de dois grupos de intervenção

nutricional, sendo que ambos os grupos visam a perda de peso numa fase de intervenção e a manutenção do peso 12 meses após o término da mesma. Não será possível a troca de grupo, mesmo que seja essa a sua intenção. Encontra uma explicação mais detalhada das duas intervenções nutricionais no seguimento deste documento.

Qual a duração esperada da sua participação?

Consoante o grupo a que seja alocada, o estudo terá a duração de 75 a 82 semanas, sendo que as primeiras 26 ou 33 semanas correspondem à fase de intervenção ou perda de peso, e as seguintes 52 semanas (12 meses) correspondem à fase de manutenção do peso perdido.

Quais os procedimentos do estudo em que vai participar?

O estudo clínico pressupõe 8 momentos de avaliação, distribuídos pelas várias fases da investigação: fase 1, 2, 3 da intervenção e fase de gestão do peso.

Na fase 1 fará apenas uma avaliação que inclui determinação do peso e avaliação da composição corporal, do consumo energético em repouso (através de calorimetria indireta) e colocação de um dispositivo que visa avaliar a sua atividade física (acelerómetro). O acelerómetro trata-se de um pequeno aparelho que é colocado na zona da anca acoplado a um cinto elástico, sendo apenas retirado para dormir e em atividades que envolvam água (por exemplo, tomar banho e nadar). Durante a sua utilização, o aparelho regista todas as acelerações do movimento, fornecendo informação sobre o número de contagens por minuto. Duas semanas depois fará nova avaliação dos mesmos parâmetros, assim como recolha de sangue (5 ml) para determinação das hormonas da tiróide T3 e T4 livre, insulina, leptina e cortisol.

Na fase 2, fará novas avaliações (exceto recolha de sangue) de 4 em 4 semanas, ou de 6 em 6 semanas, de acordo com o grupo a que foi alocada.

Na fase 3 volta a repetir estas avaliações e fazer recolha de sangue para determinar os mesmos parâmetros, e no final desta fase, passadas 7 semanas repete mais uma vez estas avaliações e recolha de sangue.

Passados 12 meses destes procedimentos repetirá as referidas avaliações e recolha de sangue. Todas as avaliações serão efetuadas após jejum noturno de pelo menos 10 horas: peso, composição corporal, consumo energético em repouso e recolha de sangue, com a exceção da avaliação da atividade física.

Nas semanas referentes às oito avaliações preconizadas no estudo, terá consulta de nutrição com um nutricionista, com vista à definição, ajuste e monitorização do plano alimentar, de acordo com os objetivos e protocolo do estudo. A restrição energética preconizada será de 33% face às necessidades energéticas diárias, sendo utilizadas estratégias nutricionais no sentido de promover uma maior saciedade e redução do apetite. O plano alimentar será individualizado, de acordo com as necessidades energéticas diárias, e terá em consideração as suas preferências alimentares. Terá como base uma alimentação do tipo mediterrânico, privilegiando os alimentos de origem vegetal, tais como hortícolas, fruta, pão e cereais pouco refinados, leguminosas, frutos secos e oleaginosas, em detrimento dos alimentos de origem animal. As confeções serão simples e o azeite será a principal fonte de gordura para cozinhar e temperar. O consumo de laticínios ou substitutos vegetais será moderado e a água será a bebida preferencial. O consumo frequente de pescado será incentivado, em contraposição ao consumo de carnes, que deverá ser reduzido, especialmente o de carnes vermelhas.

Entre estas oito avaliações/consultas pré-definidas, serão efetuados contatos adicionais via telefone a fim de acompanhar os resultados e adesão ao plano alimentar. Caso as participantes apresentem questões e dificuldades na adesão ao plano alimentar, serão prestados os esclarecimentos necessários via telefone e/ou videochamada.

Após o final da intervenção inicia-se o período de gestão de peso, que terá a duração de 12 meses, a fim de avaliar o sucesso na manutenção do peso perdido. Durante estes 12 meses a equipa de investigação continuará disponível para acompanhar as participantes à distância, com pelo menos 1 contacto mensal via telefone.

A sua participação é voluntária?

A sua participação no estudo é totalmente voluntária e pode recusar-se a integrar o mesmo. Caso decida participar neste estudo, poderá desistir do mesmo em qualquer momento, sem que isso represente uma consequência negativa para si.

Quais os possíveis benefícios da sua participação?

Considerando que o estudo inclui uma intervenção nutricional com vista à perda de peso, os benefícios identificados são: i) perda de peso e melhoria da composição corporal; ii) melhorias metabólicas decorrentes da perda de peso clinicamente significativa; iii) eventual atenuação da termogénese adaptativa, facilitando a perda e manutenção do peso perdido; iv) bem-estar e aumento da auto-confiança associadas à perda de peso; v) adoção de um estilo de vida saudável, com aprendizagem de estratégias nutricionais e aquisição de ferramentas, com vista à adoção de hábitos alimentares saudáveis e gestão eficaz do peso corporal.

Quais os eventuais riscos da sua participação?

Os eventuais riscos incluem: i) desconforto com utilização da máscara durante a avaliação de calorimetria indireta; ii) desconforto com a recolha de sangue para as determinações séricas de T3 e T4 livre, insulina, leptina e cortisol; iii) desconforto associado à utilização de um acelerómetro colocado na zona da anca durante o período de uma semana; iv) privação de alimentos de elevada densidade energética ou de qualidade nutricional insuficiente, que podem constituir uma fonte de prazer imediato para os participantes; v) tempo despendido com as visitas ao laboratório/clínica, bem como os custos associados a essas deslocações. O estudo não representará outros custos para a participante, para além dos já mencionados relativos às deslocações.

Quem assume a responsabilidade caso surja um evento negativo?

Apesar da possibilidade de ocorrer um evento negativo ser muito reduzida, caso suceda, a responsabilidade será do investigador principal.

Quem deve ser contactado em caso de urgência?

Em caso de urgência deverá contactar a investigadora Filipa Cortez, email up201908421@edu.fcna.up.pt, tel. 936587812.

Como é assegurada a confidencialidade dos dados?

A informação recolhida durante o estudo será utilizada apenas pela equipa de investigação, estando garantido o anonimato dos participantes e a confidencialidade dos dados. Os dados pessoais não estarão em discos, nem é permita a utilização de redes públicas, sendo apenas utilizadas ligações seguras encriptadas como ligações por VPN. Os documentos em papel serão guardados em sala fechada.

O que acontecerá aos dados quando a investigação terminar?

Os dados estão sob a responsabilidade da FCNAUP e serão utilizados apenas para efeitos de defesa de tese de doutoramento. Os documentos em suporte de papel serão destruídos após a construção da matriz de tratamento dos dados.

De que forma serão divulgados os resultados do estudo e com que finalidades?

Os resultados obtidos com o estudo serão tratados de forma anónima, sendo divulgados à comunidade científica através da submissão de artigos científicos com revisão por pares e presença em conferências/congressos nacionais e/ou internacionais, assim como aos participantes mediante reuniões de grupo e/ou envio de email com resultados principais.

Em caso de dúvidas quem deverei contactar?

Para qualquer questão relacionada com a sua participação neste estudo deverá contactar: Filipa Cortez, email up201908421@edu.fcna.up.pt, tel. 936587812.

Onde poderei consultar a Política de Proteção de dados deste projeto?

A política de proteção de dados deste projeto de investigação está disponível no Anexo I deste documento.

Assinatura do Consentimento Informado, Livre e Esclarecido

Declaro ter lido e compreendido a informação presente neste documento, e estou consciente do que esperar com a minha participação no presente estudo. Tive oportunidade de esclarecer todas as questões sobre a participação no estudo, e fui informada da possibilidade de livremente recusar ou abandonar a participação em qualquer momento do mesmo, sem que isso tenha consequências negativas para mim. Declaro não ter sido incluída em nenhum outro projeto de investigação nos últimos três meses. Concordo com a participação neste estudo, de acordo com os esclarecimentos que me foram prestados, como consta neste documento, do qual me foi entregue uma cópia.

Participante

Nome do participante como consta do Cartão de Cidadão

____

Cartão de Cidadão n.º ___________________________

Assinatura do participante ___________________________

Data (dd/mm/aaaa) ___________________________

Investigador/Equipa de Investigação

Nome do investigador ___________________________

Cédula Ordem dos Nutricionistas n.º ___________________________

Assinatura do investigador ___________________________

Data (dd/mm/aaaa) ___________________________
