# Supplementary material for: The BREAK study protocol: Effects of intermittent energy restriction on adaptive thermogenesis during weight loss and its maintenance
Source: PLoS One. 2023 Nov 13;18(11):e0294131. doi: 10.1371/journal.pone.0294131 (PMC10642783; doi:10.1371/journal.pone.0294131)
Supplement: S2 Appendix — (PDF) [file pone.0294131.s007.pdf]

INFORMED, FREE AND INFORMED CONSENT FOR SCIENTIFIC RESEARCH WITH HUMAN BEINGS

Study title: Effects of intermittent energy restriction on adaptive thermogenesis and successful weight loss maintenance

Project leaders: Prof. Dr. Vítor Hugo Teixeira, Prof. Dr. Analiza Mónica Silva.

This document, called **Informed, Free and Informed Consent,** contains relevant information about the clinical study you have been invited to take part in, as well as what you should expect if you decide to take part. We therefore ask you to read all the information contained here carefully, and you should feel completely free to ask questions, as well as to discuss your participation with third parties (family, friends or acquaintances), in order to decide whether to take part in this study.

General information

The research study you are being invited to take part in aims to analyze the effects of a specific nutritional intervention on the metabolic adaptations that occur during the weight loss process, as well as on the success of maintaining the weight lost. Considering that you meet the eligibility criteria, you are hereby invited to take part in the clinical study in question. It is important that you are present at the various assessment moments, following the nutritional guidelines provided in consultation as much as possible with a view to losing weight during the intervention, as well as maintaining the weight lost after the end of the weight reduction phase, over a period of 12 months.

Once you have confirmed your intention to take part by signing this document, you will be randomly allocated to one of two nutritional intervention groups, both of which aim to lose weight during an intervention phase and maintain weight 12 months after the end of the intervention. It will not be possible to change groups, even if that is your

intention. A more detailed explanation of the two nutritional interventions can be found at the end of this document.

What is the expected duration of your participation?

Depending on the group she is allocated to, the study will last between 75 and 82 weeks, with the first 26 or 33 weeks corresponding to the intervention or weight loss phase, and the following 52 weeks (12 months) corresponding to the weight loss maintenance phase.

What are the procedures for the study in which you are taking part?

The clinical study involves 8 evaluation moments, spread over the various phases of the research: phase 1, 2, 3 of the intervention and the weight management phase.

In phase 1 you will only undergo an assessment, which includes determining your weight and assessing your body composition, resting energy consumption (using indirect calorimetry) and fitting a device to assess your physical activity (accelerometer). The accelerometer is a small device that is placed on the hip area and attached to an elastic belt. It is only removed when sleeping and when doing activities that involve water (e.g. bathing and swimming). During use, the device records all the accelerations of movement, providing information on the number of counts per minute. Two weeks later, the same parameters will be assessed again, as well as blood (5 ml) taken to determine the thyroid hormones free T3 and T4, insulin, leptin and cortisol.

In phase 2, you will undergo new evaluations (except blood sampling) every 4 weeks or every 6 weeks, depending on the group you have been allocated to.

In phase 3 you repeat these assessments and take blood to determine the same parameters, and at the end of this phase, after 7 weeks, you repeat these assessments and take blood once again.

After 12 months of these procedures, they will repeat these assessments and blood draws. All assessments will be carried out after an overnight fast of at least 10 hours:

weight, body composition, resting energy consumption and blood sampling, with the exception of the physical activity assessment.

During the weeks relating to the eight assessments recommended in the study, the patient will have a nutrition consultation with a nutritionist, with a view to defining, adjusting and monitoring the diet plan, in accordance with the study's objectives and protocol. The recommended energy restriction will be 33% of daily energy needs, and nutritional strategies will be used to promote greater satiety and reduce appetite. The diet plan will be individualized, according to daily energy needs, and will take into account their food preferences. It will be based on a Mediterranean diet, favoring foods of plant origin, such as vegetables, fruit, bread and lightly refined cereals, legumes, nuts and oilseeds, to the detriment of foods of animal origin. Cooking will be simple and olive oil will be the main source of fat for cooking and seasoning. The consumption of dairy products or vegetable substitutes will be moderate and water will be the preferred drink. Frequent consumption of fish will be encouraged, as opposed to meat consumption, which should be reduced, especially red meat.

Between these eight pre-defined assessments/consultations, additional contacts will be made via telephone in order to monitor the results and adherence to the diet plan. If the participants have any questions or difficulties in adhering to the diet plan, the necessary clarifications will be provided via telephone and/or video call.

After the end of the intervention, the weight management period will begin, lasting 12 months, in order to evaluate the success in maintaining the weight lost. During these 12 months, the research team will continue to be available to monitor the participants remotely, with at least 1 monthly contact via telephone.

Is your participation voluntary?

Your participation in the study is completely voluntary and you can refuse to take part. If you decide to take part in this study, you can withdraw from it at any time, without this having any negative consequences for you.

What are the possible benefits of your participation?

Considering that the study includes a nutritional intervention aimed at weight loss, the benefits identified are: i) weight loss and improved body composition; ii) metabolic improvements resulting from clinically significant weight loss; iii) possible attenuation of adaptive thermogenesis, facilitating the loss and maintenance of the weight lost; iv) well-being and increased self-confidence associated with weight loss; v) adoption of a healthy lifestyle, with learning of nutritional strategies and acquisition of tools aimed at the adoption of healthy eating habits and effective management of body weight.

What are the possible risks of your participation?

Possible risks include: i) discomfort with wearing the mask during the indirect calorimetry assessment; ii) discomfort with blood sampling for serum determinations of free T3 and T4, insulin, leptin and cortisol; iii) discomfort associated with wearing an accelerometer placed on the hip area for the period of one week; iv) deprivation of foods of high energy density or insufficient nutritional quality, which can be a source of immediate pleasure for the participants; v) time spent visiting the laboratory/clinic, as well as the costs associated with these trips. The study will not represent any costs for the participant other than those already mentioned in relation to travel.

Who takes responsibility in the event of a negative event?

Although the possibility of a negative event occurring is very low, if it does, the principal investigator will be responsible.

Who should be contacted in an emergency?

In case of urgency, please contact the researcher Filipa Cortez, email up201908421@edu.fcna.up.pt, tel. 936587812.

How is data confidentiality ensured?

The information collected during the study will only be used by the research team, and the anonymity of the participants and the confidentiality of the data will be guaranteed. Personal data will not be on disks, nor will the use of public networks be allowed, only secure encrypted connections such as VPN connections will be used. Paper documents will be kept in a locked room.

What will happen to the data when the investigation is over?

The data is the responsibility of FCNAUP and will only be used for the purposes of defending the doctoral thesis. Paper documents will be destroyed once the data processing matrix has been constructed.

How will the results of the study be disseminated and for what purposes?

The results obtained from the study will be treated anonymously, and will be disseminated to the scientific community through the submission of peer-reviewed scientific articles and attendance at national and/or international conferences/congresses, as well as to the participants through group meetings and/or sending emails with the main results.

Who should I contact if I have any questions?

For any questions regarding your participation in this study, please contact: Filipa Cortez, email up201908421@edu.fcna.up.pt, tel. 936587812.

Where can I consult this project's Data Protection Policy?

The data protection policy for this research project is available in Annex I of this document.

Signature of Informed, Free and Informed Consent

I declare that I have read and understood the information in this document, and I am aware of what to expect from my participation in this study. I have been given the opportunity to clarify all questions regarding participation in the study, and I have been informed of the possibility of freely refusing or abandoning participation at any time during the study, without this having any negative consequences for me. I declare that I have not been included in any other research project in the last three months. I agree to take part in this study in accordance with the explanations given to me, as set out in this document, a copy of which has been given to me.

Participant

Participant's name as it appears on the Citizen Card

____

Citizen Card No. Participant's signature

____

Date (dd/mm/yyyy)

____

Researcher/Research Team

Researcher's name Cédula Ordem dos Nutricionistas no.

____

Researcher's signature Date (dd/mm/yyyy)

____
