# Supplementary material for: The BREAK study protocol: Effects of intermittent energy restriction on adaptive thermogenesis during weight loss and its maintenance
Source: PLoS One. 2023 Nov 13;18(11):e0294131. doi: 10.1371/journal.pone.0294131 (PMC10642783; doi:10.1371/journal.pone.0294131)
Supplement: Attachment — Submitted filename: Response to Reviewers.docx [file pone.0294131.s008.docx]

**Response to the editor and reviewers**

Dear Editor and Reviewers,

We would like to express our sincere appreciation for the time you devoted to this review process. Your insights and feedback have played an indispensable role in refining our work, and enhancing its quality.

**Considering the Journal Requirements**

1. Please ensure that your manuscript meets PLOS ONE's style requirements, including those for file naming.

**Reply**: We have consulted the suggested files, and proceeded as required.

2. Please amend your authorship list in your manuscript file to include authors Filipa M Cortez, Catarina L Nunes, Vítor H Teixeira, Analiza M Silva and Luís B Sardinha.

**Reply**: We changed it accordingly.

3. We note that Figure 2 in your submission contain copyrighted images. All PLOS content is published under the Creative Commons Attribution License (CC BY 4.0), which means that the manuscript, images, and Supporting Information files will be freely available online, and any third party is permitted to access, download, copy, distribute, and use these materials in any way, even commercially, with proper attribution.

**Reply**: Considering we were unable to request permission for the copyrighted images, we removed them from the submitted Figure, without affecting the understanding of its content.

4. We note that the original protocol that you have uploaded as a Supporting Information file contains an institutional logo. As this logo is likely copyrighted, we ask that you please remove it from this file and upload an updated version upon resubmission.

**Reply**: The logo was removed from the file, as required.

5. Please review your reference list to ensure that it is complete and correct. If you have cited papers that have been retracted, please include the rationale for doing so in the manuscript text, or remove these references and replace them with relevant current references. Any changes to the reference list should be mentioned in the rebuttal letter that accompanies your revised manuscript. If you need to cite a retracted article, indicate the article’s retracted status in the References list and also include a citation and full reference for the retraction notice.

**Reply**: We reviewed the reference list, and no papers have been retracted.

**Considering the Reviewers' comments**

Have the authors described where all data underlying the findings will be made available when the study is complete?

Reviewer #1: No; Reviewer #2: Yes

**Reply**: We have updated Data Availability information in the manuscript, as required.

(page 2, lines 38-45)

**Review Comments to the Author**

**Minor revision with the following remarks**
1. The introduction mentions that there are 8 randomized clinical trials (RCTs) that compare CER with IER (lines 102-104), however, only the results from studies that have found greater weight loss with IER have been detailed. It would be helpful to know the outcomes of other studies and explore potential reasons for the conflicting results.
**Reply**: We appreciate the reviewer comment, and therefore included in the text the potential reasons for the conflicting results. We did not describe the outcomes and main characteristics for the remaining studies due to length of introduction.

(page 5-6, lines 121-126)

2. The description of the REE measurement protocol, did not include information on rest time before measurement, on the calibration of the calorimeter, and the REE validity criterion (steady-state or %CV VO2).

**Reply**: We have added the required information in the manuscript.
(page 17-18, lines 376-378, 384-387)

3. As described by Nunes et al. 2022, the AT equation must be REEm -REEp (subtracting REEp from REEm), to allow the interpretation that negative values mean lower-than-expected REE due to body composition changes. In lines 365-366, the equation is inverted.
**Reply**: We have changed it accordantly.
(page 18, lines 395-396)

4. Cutoff values that define the AF intensity and the average time at each intensity level will be based on non-triaxial accelerometers (line 382)? Sasaki et al. 2011 (DOI: 10.1016/j.jsams.2011.04.003) proposed tri-axial vector magnitude (VM3) cut-points to classify physical activity (PA) intensity.

**Reply**: We appreciate the reviewer comment and would like to explain our rationale. The accelerometer allows to select the counts of the vertical axis (axis 1) to classify the levels of intensity based on Troiano's cutoff values (1). By using this option, we would be able to compare our results with other studies that used Troiano's cutoffs. Regardless, we are also able to select the vector magnitude based on the 3 axis and classify the level of intensity based on Freedson cut-points. Considering the reviewer's point based on the paper of Sasaki et al. 2011, we changed the cutoff values for those proposed by these authors (2).

(page 19, lines 416-419)

7. PLOS authors have the option to publish the peer review history of their article. If published, this will include your full peer review and any attached files.

**Reply**: We opt to publish peer-reviewed history of the article.

**References**

1.	Troiano RP, Berrigan D, Dodd KW, Mâsse LC, Tilert T, McDowell M. Physical activity in the United States measured by accelerometer. Med Sci Sports Exerc. 2008;40(1):181-8.

2.	Sasaki JE, John D, Freedson PS. Validation and comparison of ActiGraph activity monitors. J Sci Med Sport. 2011;14(5):411-6.
